# Maximizing the effectiveness of 1.5 mg levonorgestrel for emergency contraception: The case for precoital use^☆^

**DOI:** 10.1016/j.conx.2024.100107

**Published:** 2024-05-18

**Authors:** Douglas J. Taylor, Nathalie Kapp, Markus J. Steiner

**Affiliations:** a FHI 360, Product Development and Introduction Department, Durham, NC, United States; b International Planned Parenthood Federation, London, UK

**Keywords:** Emergency contraception, Levonorgestrel, Mathematical modeling, Unintended pregnancy

## Abstract

**Objectives:**

U.S. and World Health Organization Selected Practice Recommendations for Contraceptive Use state people may have an advanced supply of emergency contraception (EC) to minimize treatment delays. We sought to characterize the potential improvement in effectiveness of 1.5 mg levonorgestrel (LNG-EC) if it were taken up to a few hours before unprotected sex.

**Study design:**

We expanded on an existing mathematical model for the maximum attainable effectiveness of LNG-EC, assuming it exclusively works to disrupt ovulation, and compared results with point estimates from nine studies when it was taken up to 72 hours after sex. We then modelled how effectiveness might have improved if subjects had taken LNG-EC up to 3 hours before sex.

**Results:**

Taking LNG-EC immediately after sex could potentially reduce the risk of unintended pregnancy by 91%. However, population-average maximum attainable effectiveness levels ranged from just 49% to 67% when accounting for the distributions of postcoital treatment delays in the example studies. If half the subjects had taken it 3 hours before sex, then maximum effectiveness levels would have ranged from 70% to 81%.

**Conclusions:**

At the individual level, taking LNG-EC a few hours before sex is a logical extension of Selected Practice Recommendations regarding an advanced supply of EC and, based on our modeling, should be advocated for people who can reasonably anticipate an unprotected sex act. In the absence of more clinical data, however, people should not routinely rely on precoital use of LNG-EC to prevent pregnancy unless modern, effective contraceptives are inaccessible to them.

**Implications:**

Based on mathematical modeling, individuals who anticipate needing to take LNG-EC for an impending unprotected act of sex could further reduce their chance of an undesired pregnancy by taking it a few hours in advance.

## 1 Introduction

A 1.5 mg oral dose of levonorgestrel (LNG) is a generally safe and effective emergency contraceptive (EC) when ingested before ovulation but has no apparent impact on the risk of pregnancy when taken afterward [1]. Based on published data from two trials that carefully timed ovulation using follicle size and/or hormone data, 0 of 137 subjects who had unprotected sex in their fertile period (the 6 days leading up to and including ovulation) and ingested LNG-EC before ovulation became pregnant, while 11 of 62 (17.7%) who took the drug after ovulation became pregnant [2], [3]. These results underscore LNG-EC’s primary method of action, which is to interfere with ovulatory function by blunting or delaying the preovulatory luteinizing hormone (LH) surge for longer than the 5 days sperm may remain viable in the female reproductive tract [4], [5]. The need to take LNG-EC prior to ovulation, however, means that its typical effectiveness decreases with the delay to ingestion of the drug; hence, the regulatory guidance that it be taken as soon as possible within 72 hours after sex [6].

An advanced supply of LNG-EC has the potential to reduce treatment delays and increase effectiveness. In one randomized trial, people who received advanced provision and unlimited resupply of LNG-EC were twice as likely to ever take it than in a postcoital access group, and the median delay to ingestion was 24 hours shorter [7]. The advanced provision did not translate into a lower cumulative (12-month) incidence of pregnancy, however, possibly due to differential substitution of EC for other contraceptive methods [8]. Since the 1970s, investigators have explored the related question of whether LNG has utility as a routine “on-demand” contraceptive pill taken shortly before or after sex. An extensive summary of the evidence for such peri-coital use is provided in a 2014 Cochrane review [9]. More recently, a multicountry study in which individuals were instructed to take 1.5 mg LNG within 24 hours before or after sex reported a Pearl Index of 10.3 pregnancies per 100 person-years among those aged 18 to 35 years, but the precision of the estimate was modest (95% confidence interval [CI]: 5.4–19.9) [10].

In this paper, we review challenges associated with estimating the effectiveness of LNG-EC and revisit an alternative model-based measure, the maximum attainable effectiveness of LNG-EC given its method of action, that avoids certain limitations of clinical study data. We then model the extent to which the effectiveness of LNG-EC might be increased when it is available in advance, including taking it a few hours before sex if an act would otherwise go unprotected. Such precoital use is a logical extension of U.S. and World Health Organization (WHO) Selected Practice Recommendations for Contraceptive Use that people have an advanced supply so that it can be used “as soon as possible after unprotected intercourse” [11], [12].

## 2 Materials and methods

### 2.1 Historical approaches to estimating effectiveness of EC

The per-act effectiveness of EC is expressed as

(1) $$1-P_{T}/P_{\mathrm{NT}},$$

where *P*_*T*_ is the probability of pregnancy following treatment and *P*_*NT*_ is the probability had—counter to fact—treatment not been received. In practice, *P*_*T*_ is estimated by the proportion of subjects in a study who become pregnant despite using EC and *P*_*NT*_ by the average of each subject’s counterfactual risk of pregnancy. As a point of emphasis, Equation 1 is the relative risk reduction for a single act of sex when EC is definitively used and not a measure of absolute risk (e.g., a Pearl Index or cumulative probability of pregnancy) during routine use of a contraceptive method.

Most EC studies have assumed there is a nonzero probability of clinical pregnancy due to unprotected sex in a 6-day fertile window when computing each subject’s contribution to *P*_*NT*_. These probabilities were estimated by Wilcox et al. [13] as 0.04, 0.13, 0.08, 0.29, 0.27, and 0.08 for days −5 to 0 relative to ovulation, respectively; sex on any other day is presumed to carry essentially no risk. The difficulty lies in determining each person’s day of ovulation absent EC use. The earliest approach was to assume everyone would have ovulated 14 days prior to their next expected menses; here, only people who had sex 19 to 14 days prior to their next menses contribute a nonzero probability to *P*_*NT*_. In addition to the challenge of estimating next menses based on recall of previous menses and typical cycle length, the implicit assumption that luteal phase lengths do not vary within or between individuals is inaccurate and likely leads to overestimating *P*_*NT*_ [14]. Noting this limitation, subsequent investigators combined an empirical distribution of follicular phase lengths with the Wilcox probabilities, resulting in a nonzero, cycle-day-specific probability of pregnancy contributed to *P*_*NT*_ by essentially every study subject [15], [16]. Although statistically rigorous, individuals still need to recall the date of their previous menses to determine the cycle day on which sex occurred. A third, more precise approach is to estimate each person’s day of ovulation based on follicular and hormone data at the time they present for EC, which avoids the need to know the date of last menses [2], [3].

Even if ovulation days were known exactly, an effectiveness estimate could be biased because the Wilcox probabilities were derived from couples without a history of fertility problems who were trying to conceive and excluded cycles where ovulation was not detected. In contrast, studies of EC involve subjects actively trying to avoid pregnancy and for whom fertility status is less certain. Similarly, people may present for EC despite partial or complete protection from another method, such as condom failure during withdrawal or maintenance of therapeutic drug levels after having stopped use of a routine hormonal method [17], [18]. Both subfertility and protection from another method would lead to overestimating *P*_*NT*_ and the effectiveness of EC. On the other hand, *P*_*NT*_ could be underestimated if subjects had additional acts of sex around the time that they used EC [19]. Given these uncertainties, it is not surprising that effectiveness estimates in cohorts taking 1.5 mg LNG-EC within 72 hours after unprotected sex vary widely (from 52% to 97% based on our review of the literature [3], [20], [21], [22], [23], [24], [25], [26], [27]).

### 2.2 Maximum attainable effectiveness

Previous investigators have considered the maximum attainable effectiveness of EC, assuming it prevents pregnancy 100% of the time when taken before ovulation but has no effect when taken afterward. Based on their findings, Trussell and Raymond [28] and Mikolajczyk and Stanford [29], respectively, made compelling arguments that Yuzpe (an EC regimen containing both LNG and ethinyl estradiol) and LNG-EC were unlikely to be as effective as indicated in reports available at the time if their only method of action was to disrupt ovulation. However, more recent studies (and reanalysis of older studies) using refined methods to compute counterfactual pregnancy risks have obtained effectiveness estimates more in line with those investigators’ maximum attainable values [3], [14], [26], [27], and it is now generally accepted that “data are weak to speculative regarding any postovulatory mechanistic effects” for LNG-EC [30]. Regardless of the historical context, important insights can still be gained by considering the concept of maximum attainable effectiveness.

Mikolajczyk and Stanford [29] used simulations to estimate the maximum effectiveness of LNG-EC as a function of treatment delay based on a model of follicular size at the time of drug use. Here, we generalize the discrete probability model of Trussell and Raymond [28] by incorporating nonuniform probabilities of sex in the fertile period and variable treatment delays. Recall that a nonzero risk of pregnancy is presumed to exist only if sex occurs on one of the 6 days leading up to and including ovulation. Thus, taking LNG-EC one or more days before sex would mean it was ingested at least a day before ovulation for any act that could lead to pregnancy, and its potential effectiveness is 100%. Alternatively, if LNG-EC is taken more than 5 days after sex, then it is not ingested before ovulation for any act where risk exists, and its effectiveness is 0%. In between these extremes, when it is taken *J* = 0 to 5 days after sex, the maximum attainable effectiveness of LNG-EC is

(2) $$E_{\max,J}=1-\left[\left(\sum_{i=-J}^{0}\pi_{i}\cdot P_{i}\right)/\left(\sum_{i=-5}^{0}\pi_{i}\cdot P_{i}\right)\right].$$

Here, *π*_*i*_ is the probability that a sex act in the fertile period occurs on the day *i* = −5 to 0 relative to ovulation, and *P*_*i*_ is the associated Wilcox probability of pregnancy.

The denominator in Equation 2 is the probability of pregnancy if sex occurs in the fertile period but LNG-EC is not used, and the numerator is the corresponding probability given treatment. The latter presumes LNG-EC prevents pregnancy, but only if taken one or more days before ovulation and regardless of any subject-level factors (e.g., obesity) that could negatively impact effectiveness [31], [32]. Hence the expression “maximum attainable,” even if it may not be realized for all individuals. Additional details of Equation 2 are provided in the Supplementary Appendix, but we emphasize three points here. First, it is not a function of cycle day when sex occurs (a source of recall bias in clinical studies). Second, the solution would not change if the *P*_*i*_ were systematically overestimating counterfactual risks in a population trying to avoid pregnancy (another source of bias with clinical data). And third, the maximum attainable effectiveness in a cohort ingesting a drug at differing times can be computed as a weighted average of $E_{\max,J}$ values, with weights determined by the distribution of treatment delays.

## 3 Results

### 3.1 Maximum attainable effectiveness as a function of treatment delay

**Fig. 1:**
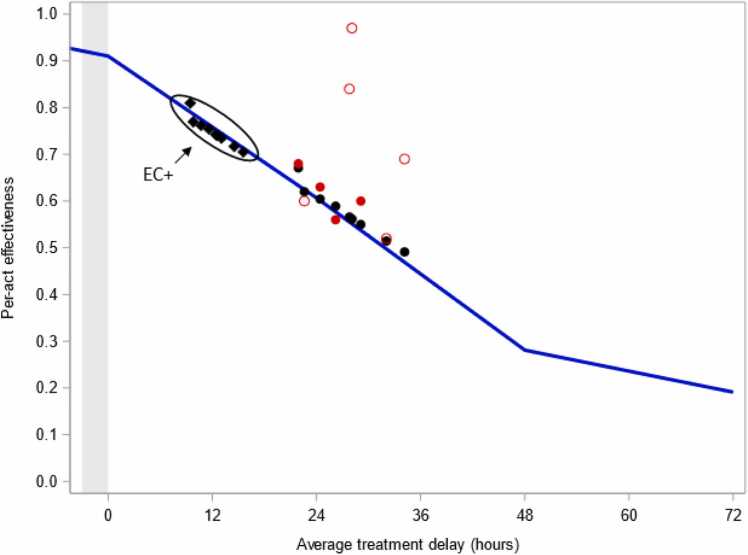
Maximum attainable per-act effectiveness of levonorgestrel emergency contraceptive as a function of treatment delay (blue line) and when accounting for the distributions of treatment delays in nine studies published between 1993 and 2023 (black circles, plotted versus the estimated mean delay in each cohort [3], [20], [21], [22], [23], [24], [25], [26], [27]). Solid red circles are point estimates of effectiveness from studies where counterfactual risks of pregnancy could be computed based on cycle-day-specific conception probabilities or follicle size and hormone data, and open red circles are point estimates that assume ovulation occurs exactly 14 days prior to the next expected menses. Black diamonds (grouped as “EC+”) are hypothetical maximum attainable values if 50% of subjects in each study had an advanced supply of levonorgestrel emergency contraceptive and took it three hours before sex (denoted by gray-shaded region). Results assume unprotected sex is equally likely to occur on each fertile day unless an alternate empirical distribution could be derived from the study report [3]. EC = emergency contraceptive.

Solving Equation 2 requires knowing the distribution of sex acts within the fertile period. We initially assume these are uniformly distributed (i.e., *π*_*i*_ = 1/6 for each fertile day), which closely agrees with the predictions of Li et al. [16] when deriving their cycle-day-specific pregnancy probabilities. Here, the maximum attainable effectiveness of LNG-EC is 91% when it is taken immediately after sex and decreases rapidly thereafter to 61%, 28%, and 19%, respectively, when taken 1, 2, or 3 days later (Fig. 1, blue line). These are within three percentage points of the corresponding values obtained by Mikolajczyk and Stanford [29] when they assumed preovulatory use of LNG-EC is 100% effective at preventing fertilization.

We next explore the potential impact of deviations from the uniform distribution assumption. Based on follicle size and hormone data in Noé et al. [3], we estimated sex in the fertile period was most common 2 days before (*π*_−__2_ = 0.26) and least common 5 days before (*π*_−__5_ = 0.10) ovulation. This substantial deviation from a uniform distribution nonetheless leads to similar maximum effectiveness levels: 94% when LNG-EC is taken right after sex and 68%, 23%, and 13% when taken 1, 2, or 3 days later. Similarly, Novikova et al. [2] determined that sex was most common 3 days before ovulation (*π*_−__3_ = 0.24) and least common on the day of ovulation (*π*_0_ = 0.12). Here again, maximum effectiveness levels are consistent with those obtained under a uniform distribution assumption (see Supplementary Appendix for details).

### 3.2 Maximum attainable effectiveness in cohorts of subjects

In clinical studies, the postcoital delay to EC is not fixed but varies across subjects. Accordingly, we explored the population-average maximum attainable effectiveness of LNG-EC in nine studies where a point estimate of effectiveness was available or could be derived from the published findings, restricted to subjects taking the drug within 72 hours after sex. This included six studies where subjects received a single 1.5 mg dose of LNG [3], [22], [24], [25], [26], [27] and three where subjects received two 0.75 mg doses at 12-hour intervals [20], [21], [23], which were shown to be comparably effective regimens in two randomized trials [22], [24]. To be included in our analysis, a study also had to provide sufficient details to allow us to estimate a continuous (truncated-Weibull) distribution of treatment delays, such as the percentage of subjects taking EC in 24-hour intervals (see Supplementary Appendix for details). Finally, we assumed sex acts were equally likely to occur on each day in the fertile period unless the study detailed otherwise.

Maximum attainable effectiveness levels ranged from 49% to 67% when accounting for the distribution of treatment delays in the nine studies (Fig. 1, black circles; Table 1). These were within five percentage points of estimates from the four studies where cycle-day-specific conception probabilities or hormone and follicle size data were used (or could be retrospectively applied) to compute counterfactual pregnancy risks (Fig. 1, solid red circles) [3], [21], [26], [27]. In contrast, the point estimates were up to 41% greater than our maximum attainable values in the studies that used fixed luteal phase lengths to compute counterfactual pregnancy risks (Fig. 1, open red circles), notably the one that informed prescribing information for LNG-EC (point estimate of 84% versus maximum attainable of 56%) [6], [22].

**Table 1:** Effectiveness estimates and maximum obtainable values for studies published between 1993 and 2023 that reported on subjects using LNG-EC up to 72 hours after unprotected sex

Study	*N*	Mean (IQR) delay to EC^a^	Point Estimate^b^	Maximum attainable^c^	Maximum if 50% took EC 3 h before sex^c^, ^d^
Ho and Kwan (1993) [20]^e^, ^f^	410	23 (12–33)	0.60^j^	0.62	0.77
von Hertzen et al. (2002) [22]	1198	28 (13–40)	0.84^j^	0.56	0.74
Creinin et al. (2006) [23]^e^	774	34 (20–48)	0.69^j^	0.49	0.70
Dada et al. (2010) [24]	1257	28 (14–40)	0.97^j^	0.56	0.74
Glasier et al. (2010) [25]^f^	852	32 (18–45)	0.52^j^	0.51	0.72
WHO (1998) [21], [33]^e^	974	29 (15–41)	0.85^j^	0.55	0.73
Secondary analysis of WHO (1998)^g^	-	-	0.60^k^	-	-
Leung et al. (2016) [26]^h^	4470	26 (10–40)	0.56^k^	0.59	0.75
Li et al. (2023) [27]	418	24 (12–34)	0.63^k^	0.60	0.76
Noé et al. (2011) [3]^i^	393	22 (9–31)	0.68^l^	0.67	0.81

EC, emergency contraceptive; IQR, interquartile range; WHO, World Health Organization.

*N* = number of subjects included in analysis.

a Delay to EC (in hours) estimated based on information in the study reports. See Supplementary Appendix for details.

b Point estimates were extracted directly from the individual reports unless otherwise indicated.

c Assuming sex was equally likely to occur on any fertile day unless the study detailed otherwise.

d Assuming the other 50% had no change in their treatment delay.

e Study results are for two 0.75 mg doses of LNG taken at 12-hour intervals.

f Point estimate computed based on observed and expected number of pregnancies described in the report.

g Primary effectiveness result was based on fixed luteal phase lengths, but an estimate based on cycle-day-specific conception probabilities could be derived based on a secondary data source [14]. See Supplementary Appendix for details.

h Study did not indicate maximum treatment delay. However, based on the reported mean and standard deviation, nearly all subjects took it within 72 hours of unprotected sex.

i Although not explicitly restricted to 72 hours, the treatment delay exceeded 72 hours in <2% of subjects.

j Ovulation estimated as next menses minus 14 days.

k Using cycle-day-specific probabilities to compute counterfactual risks of pregnancy.

l Ovulation estimated based on hormone and ultrasound data.

U.S. and WHO Selected Practice Recommendations state that individuals may receive an advance supply of EC so it is available when needed, and we can explore the impact of this recommendation using Equation 2. For example, based on our analysis of results in Raymond et al. [7], the mean (interquartile range) treatment delay was 17 hours (4–24) with advanced provision of LNG-EC versus 32 hours (17–45) with standard postcoital access among doses taken within 72 hours after sex. Given these distributions, the maximum attainable per-act effectiveness was 66% in the advanced provision group and 52% for standard of care. These results make it apparent that an advanced supply may not dramatically increase population-average effectiveness if a large percentage of individuals nonetheless delay its use for many hours. However, if 50% of people had taken LNG-EC 3 hours before sex (and the remainder took it postcoitally as usual), maximum attainable effectiveness levels in the nine illustrative studies would have ranged from 70% to 81% (Fig. 1, black diamonds; Table 1).

## 4 Discussion

Clinical studies that precisely time ovulation in relation to LNG-EC use have shown that the method can be highly effective when it is ingested before ovulation but has no impact when taken afterward [2], [3]. Based on this simple relationship, we modeled the maximum attainable effectiveness of LNG-EC for different patterns of treatment delays described in the literature. Our results emphasize that the population-average effectiveness of LNG-EC is unlikely to exceed 67% because the drug is frequently ingested too late to interfere with ovulation.

Facilitating an advanced supply of LNG-EC could reduce treatment delays and improve effectiveness. Based on our modeling, however, population-average effectiveness would not have exceeded 81% even if half of all people in the example studies had been able and motivated to take it a few hours before sex. Recognizing that most people may not know they need EC until after the fact, even 81% may not be realistic. Among the subset of individuals who can anticipate fully unprotected sex, however, the effectiveness of LNG-EC could exceed 90% when taken within a few hours before or after intercourse. The absolute risk of pregnancy if LNG-EC was routinely used this way is beyond what can be addressed with our model. Noting that the pregnancy rate was significantly greater than 5 per 100 person-years in a proof-of-concept study of such a regimen [10], it is unlikely to be as effective as many modern methods. Co-treatment with a nonsteroidal anti-inflammatory agent might improve the effectiveness of LNG-EC [27], [34] and could prove to be a better “on-demand” contraceptive option pending future research.

Our model does not implicitly assume that sex acts among LNG-EC users convey the same pregnancy risk as in a population trying to conceive. It also avoids the need to recall the cycle day on which unprotected sex took place. The latter is replaced by an assumption about the distribution of sex acts in the fertile period, one that does not appear overly restrictive based on sensitivity analyses. It remains possible, however, that our maximum attainable values substantially overestimate real-world effectiveness. For example, our model does not account for the potential detrimental impact of obesity on the effectiveness of LNG-EC [31], [32]. We also assumed LNG-EC remains 100% effective when taken just 1 day before ovulation. Although pharmacodynamic studies have demonstrated that the method can be highly effective at disrupting ovulation when taken prior to the LH surge, the surge generally peaks one or more days before ovulation. Hence, even our modest maximum effectiveness levels of 49% to 67% in the example studies may be optimistic. Indeed, if LNG-EC had no impact when ingested within 2 days of ovulation (after the LH surge has typically begun), then we would expect effectiveness to be less than 40%. Previous investigators (including DT and MS) estimated the minimum effectiveness of LNG-EC to be 49% (95% CI: 17%–69%) based on randomized comparisons to the Yuzpe EC regimen [18]. More recently, LNG-EC was associated with 36% lower adjusted odds of pregnancy (95% CI: 0.13–0.53) in a population-based study [26]. Viewed in this light (and assuming Yuzpe does not increase the risk of pregnancy), our maximum effectiveness levels appear reasonable. Although evidence for secondary mechanisms of action is considered weak, it remains conceivable that LNG-EC impacts the risk of fertilization when ingested before ovulation but too late to blunt the LH surge, which could help explain the lack of pregnancies among individuals treated in this brief preovulatory window in at least one trial [3].

The U.S. and WHO Selected Practice Recommendations for Contraceptive Use include statements that people may have a supply of EC in advance of unprotected sex so that it can be used as soon as possible after intercourse. Given the apparent rapid drop-off in the typical effectiveness of LNG-EC with postcoital treatment delay, taking it 2 to 3 hours before sex should be an option for individuals who anticipate what would otherwise be an unprotected act of sex due, for example, to the inability to negotiate condom use or gaps in adherence to regular hormonal methods. In the absence of more clinical data, however, sexually active people should not routinely rely on precoital LNG-EC to prevent pregnancy if highly effective alternatives like intrauterine devices and implants are available and acceptable to them.

## Declaration of Competing Interest

The authors receive grant funding from nonprofit foundations to support research on contraceptive methods, including 1.5 mg levonorgestrel for routine (nonemergency) use. None of these relationships have inappropriately biased this work on emergency contraception.

## Footnotes

Supplementary material associated with this article can be found in the online version at doi:10.1016/j.conx.2024.100107.

## References

[1] Endler M., Li R.H.W., Danielsson K.G. (2022). Effect of levonorgestrel emergency contraception on implantation and fertility: a review. Contraception.

[2] Novikova N., Weisberg E., Stanczyk F., Croxatto H.B., Fraser I.S. (2007). Effectiveness of levonorgestrel emergency contraception given before or after ovulation - a pilot study. Contraception.

[3] Noé G., Croxatto H.B., Salvatierra A.M., Reyes V., Villarroel C., Muñoz C. (2011). Contraceptive efficacy of emergency contraception with levonorgestrel given before or after ovulation. Contraception.

[4] Croxatto H.B., Brache V., Pavez M., Cochon L., Forcelledo M.L., Alvarez F. (2004). Pituitary-ovarian function following the standard levonorgestrel emergency contraceptive dose or a single 0.75-mg dose given on the days preceding ovulation. Contraception.

[5] Brache V., Cochon L., Deniaud M., Croxatto H.B. (2013). Ulipristal acetate prevents ovulation more effectively than levonorgestrel: analysis of pooled data from three randomized trials of emergency contraception regimens. Contraception.

[6] U.S. Food and Drug Administration. Plan B One-Step: highlights of prescribing information. https://www.accessdata.fda.gov/drugsatfda_docs/label/2009/021998lbl.pdf (accessed May 14, 2024).

[7] Raymond E.G., Stewart F., Weaver M., Monteith C., Van Der Pol B. (2006). Impact of increased access to emergency contraceptive pills. A randomized controlled trial. Obstet Gynecol.

[8] Weaver M.A., Raymond E.G., Baecher L. (2009). Attitude and behavior effects in a randomized trial of increased access to emergency contraception. Obstet Gynecol.

[9] Halpern V., Raymond E.G., Lopez L.M. (2014). Repeated use of pre- and postcoital hormonal contraception for prevention of pregnancy. Cochrane Database Syst Rev.

[10] Festin M.P.R., Bahamondes L., Nguyen T.M.H., Habib N., Thamkhantho M., Singh K. (2016). A prospective, open-label, single arm, multicentre study to evaluate efficacy, safety and acceptability of pericoital oral contraception using levonorgestrel 1.5 mg. Hum Reprod.

[11] Curtis K.M., Jatlaoui T.C., Tepper N.K., Zapata L.B., Horton L.G., Jamieson D.J. (2016). U.S. selected practice recommendations for contraceptive use, 2016. MMWR Recomm Rep.

[12] World Health Organization (2016). Selected practice recommendations for contraceptive method use.

[13] Wilcox A.J., Weinberg C.R., Baird D.D. (1998). Post-ovulatory ageing of the human oocyte and embryo failure. Hum Reprod.

[14] Trussell J., Ellertson C., von Hertzen H., Bigrigg A., Webb A., Evans M. (2003). Estimating the effectiveness of emergency contraceptive pills. Contraception.

[15] Wilcox A.J., Dunson D.B., Weinberg C.R., Trussel J., Baird D.D. (2001). Likelihood of conception with a single act of intercourse: providing benchmark rates for assessment of post-coital contraceptives. Contraception.

[16] Li D., Wilcox A.J., Dunson D.B. (2015). Benchmark pregnancy rates and the assessment of postcoital contraceptives: an update. Contraception.

[17] Jensen J.T., Edelman A., Westhoff C.L., Schreiber C.A., Archer D.F., Teal S. (2024). Use of serum evaluation of contraceptive and ovarian hormones to assess reduced risk of pregnancy among women presenting for emergency contraception in a multicenter clinical trial. Contraception.

[18] Raymond E., Taylor D., Trussell J., Steiner M.J. (2004). Minimum effectiveness of the levonorgestrel regimen of emergency contraception. Contraception.

[19] Stirling A., Glasier A. (2002). Estimating the efficacy of emergency contraception – how reliable are the data?. Contraception.

[20] Ho P.C., Kwan M.S. (1993). A prospective randomized comparison of levonorgestrel with the Yuzpe regimen in post-coital contraception. Hum Reprod.

[21] WHO Task Force on Postovulatory Methods of Fertility Regulation (1998). Randomized controlled trial of levonorgestrel versus the Yuzpe regimen of combined oral contraceptives for emergency contraception. Lancet.

[22] von Hertzen H., Piaggio G., Din J., Chen J., Song S., Bártfai G. (2002). Low dose mifepristone and two regimens of levonorgestrel for emergency contraception: a WHO multicentre randomised trial. Lancet.

[23] Creinin M.D., Schlaff W., Archer D.F., Wan L., Frezieres R., Thomas M. (2006). Progesterone receptor modulator for emergency contraception: a randomized controlled trial. Obstet Gynecol.

[24] Dada O.A., Godfrey E.M., Piaggio G., von Hertzen H., On behalf of the Nigerian Network for Reproductive Health Research and Training (2010). A randomized, double-blind, noninferiority study to compare two regimens of levonorgestrel for emergency contraception in Nigeria. Contraception.

[25] Glasier A., Cameron S.T., Fine P.M., Logan S., Casale W., Van Horn J. (2010). Ulipristal acetate versus levonorgestrel for emergency contraception: a randomised non-inferiority trial and meta-analysis. Lancet.

[26] Leung V., Soon J.A., Lynd L.D., Marra C.A., Levine M. (2016). Population-based evaluation of the effectiveness of two regimens for emergency contraception. Int J Gynaecol Obstet.

[27] Li R.H.W., Lo S.S.T., Gemzell-Danielsson K., Fong C.H.Y., Ho P.C., Ng E.H.Y. (2023). Oral emergency contraception with levonorgestrel plus piroxicam: a randomised double-blind placebo-controlled trial. Lancet.

[28] Trussell J., Raymond E.G. (1999). Statistical evidence about the mechanism of action of the Yuzpe regimen of emergency contraception. Obstet Gynecol.

[29] Mikolajczyk R.T., Stanford J.B. (2007). Levonorgestrel emergency contraception: a joint analysis of effectiveness and mechanism of action. Fertil Steril.

[30] U.S. Food and Drug Administration. Levonorgestrel 1.5 mg Tablet Emergency Contraceptive. Labeling Supplement for Update to Mechanism of Action Information; 2022, p.1. https://www.accessdata.fda.gov/drugsatfda_docs/label/2022/021998Orig1s005SumR.pdf (accessed May 14, 2024).

[31] Glasier A., Cameron S.T., Blithe D., Scherrer B., Mathe H., Levy D. (2011). Can we identify women at risk of pregnancy despite using emergency contraception? Data from randomized trials of ulipristal acetate and levonorgestrel. Contraception.

[32] Kapp N., Abitbol J.L., Mathé H., Scherrer B., Guillard H., Gainer E. (2015). Effect of body weight and BMI on the efficacy of levonorgestrel emergency contraception. Contraception.

[33] Piaggio G., von Hertzen H., Grimes D.A., Van Look P.F.A., On behalf of the Task Force on Postovulatory Methods of Fertility Regulation (1999). Timing of emergency contraception with levonorgestrel or the Yuzpe regimen. Lancet.

[34] Massai M.R., Forcelledo M.L., Brache V., Tejada A.S., Salvatierra A.M., Reyes M.V. (2007). Does meloxicam increase the incidence of anovulation induced by single administration of levonorgestrel in emergency contraception? A pilot study. Hum Reprod.

