# Supplementary material for: Maximizing the effectiveness of 1.5 mg levonorgestrel for emergency contraception: The case for precoital use
Source: Contracept X. 2024 May 18;6:100107. doi: 10.1016/j.conx.2024.100107 (PMC11214990; doi:10.1016/j.conx.2024.100107)
Supplement: Supplementary file 1 — Supplementary material [file mmc1.docx]

**Supplemental Appendix**

In this appendix we provide additional details of the methods and data sources used in the paper.

**1.0 Derivation of Manuscript Eq. (2)**

The effectiveness of LNG-EC is defined as 1 minus the relative risk (RR) of pregnancy, where RR is the ratio of the population average probability of pregnancy given treatment with EC (*P_T_*) to the population average probability of pregnancy were - counter to truth - EC not taken (*P_NT_*). Assume pregnancy can only occur when unprotected sex takes place in the 6-day fertile period leading up to and including ovulation. Then *P_NT_* equals the probability sex occurred in the fertile period (*P_F_*) times the probability of pregnancy given sex took place in the fertile period. The latter is the weighted sum

$\sum_{i=-5}^{0} \pi_{i}\cdot P_{i} ,$

where *P_i_* is the Wilcox probability of pregnancy if sex occurs on day *i* relative to ovulation (*i* = -5 to 0), the weight *π_i_* is the probability that a sex act in the fertile period occurred on day *i*, and $\sum_{i=-5}^{0} \pi_{i}=1$. For example, if sex is equally likely to occur on any fertile day, then *π_i_* = 1/6 for all *i* and the probability of pregnancy due to an act in the fertile period is (1/6)∙(0.04+0.13+0.08+0.29+0.27+0.08)=0.15.

Next consider the conditional probability of pregnancy given a sex act occurs in the fertile period, LNG-EC is used, and LNG-EC is 100% effective at preventing pregnancy when taken one or more days before ovulation. As above, the conditional probability of pregnancy given sex occurred in the fertile period is a weighted sum of probabilities. However, the Wilcox probability (*P_i_*) is replaced by zero if the treatment delay is less than the absolute value of *i*. For example, if EC is taken immediately after sex, then only acts that occur on the day of ovulation can lead to pregnancy and the weighted sum is *π_0_*∙*P_0_*; if EC is taken one day after sex, then only acts that occur one day before ovulation or on the day of ovulation can lead to pregnancy, and the weighted sum is *π_0_*∙*P_0_* + *π_-1_*∙*P_-1_*; etc. More generally, if EC is taken *J* days after sex, then the probability of pregnancy given sex occurred in the fertile period is

$\sum_{i=-j}^{0} \pi_{i}\cdot P_{i} .$

We now derive the maximum attainable effectiveness of LNG-EC when taken *J* = 0 to 5 days after sex as

$E_{max,J}=1-\left[ \left( \sum_{i=-J}^{0} \pi_{i}\cdot P_{i} \right)/\left( \sum_{i=-5}^{0} \pi_{i}\cdot P_{i} \right) \right] ,$ (Equation A.1)

where the denominator in brackets is the probability of pregnancy if sex occurs in the fertile period but EC is not used, and the numerator is the corresponding probability given treatment.

Now imagine EC users are on average less fertile or partially protected from another contraceptive method in comparison to the population used to estimate the *P_i_* values by Wilcox *et al.* [13]. If the typical reduction (*R*) in counterfactual pregnancy risk is consistent across fertile days, then we can replace each $P_{i}$ with $P_{i}^{*}={RP}_{i}$. But then *R* cancels out from the numerator and denominator of Eq. (A.1), so that the result does not change. The same would not hold, however, if *R* is not consistent across fertile days. For example, if individuals are more or less likely to use a condom depending on which of the 6 fertile days they have sex, then partial protection from condom failure would vary by fertile day.

**2.0 Estimating the Probability of Sex on Each Fertile Day**

The solution to Eq. (A.1) requires knowing, or making a valid assumption about, the probability that a sex act in the fertile period occurs on each individual fertile day (*π_i_*, *i* =-5 to 0). We evaluated three data sources when assessing this distribution:

1. Li *et al*. [16] predicted the probability that a single sex act occurs on each of days -5 to 0 relative to ovulation to be 0.042, 0.039, 0.039, 0.041, 0.040, and 0.045, respectively, when modelling cycle-day specific probabilities of pregnancy. Dividing each of these by the overall probability that sex occurs somewhere in the fertile period (0.247), we obtain the following near uniform distribution: *π_-5_* = 0.170, *π_-4_* = 0.158, *π_-3_* = 0.158, *π_-2_* = 0.166, *π_-1_* = 0.162, and *π_0_* = 0.182.
2. Noé *et al*. [3] provided the number of subjects (out of N=148) who had sex during the fertile period in half-day intervals in Figure 3 of their report. For subjects who did not fall on an integer day, we assigned a weight of 0.5 to both the previous and next day and obtained the following distribution: *π_-5_* = 0.101, *π_-4_* = 0.145, *π_-3_* = 0.203, *π_-2_* = 0.264, *π_-1_* = 0.166, and *π_0_* = 0.122.
3. Novikova *et al*. [2] provided the numbers of subjects (out of N=51) predicted to have had sex on each fertile day in Table 1 of their report. We used these counts to estimate the following distribution: *π_-5_* = 0.137, *π_-4_* = 0.176, *π_-3_* = 0.235, *π_-2_* = 0.118, *π_-1_* = 0.216, and *π_0_* = 0.118. Using these parameters in Eq. (A.1) results in maximum effectiveness levels of 94%, 55%, 32%, and 19% if LNG-EC is ingested 0, 1, 2, and 3 days after sex (results not shown in the paper).
4. **3.0 Maximum Effectiveness in a Cohort of Subjects**

Consider a cohort of subjects who ingest EC at varying hours *t* after sex according to the cumulative probability distribution function *F(t)*. Then the maximum attainable effectiveness of LNG-EC in the cohort is computed by integrating $E_{max,J}$ over the distribution of treatment delays. We approximated this expectation by taking a weighted average of $E_{max,J}$ values, with weights determined by the probability that subjects ingest EC in 1-hour intervals (ω_t_ *= F(t) – F(t-1)*, 1 < *t* ≤ 72).

**3.1 Choice of Parametric distribution for Treatment Delays**

Most clinical studies report treatment delays in 24 hours intervals, which is too coarse to distinguish among plausible choices for *F(t)*. However, a secondary analysis of the 1998 WHO study reported treatment delays in finer, 12-hours intervals [21,22]. We evaluated two parametric distributions for interval-censored time to event outcomes for these data: lognormal and Weibull. Each model was right truncated at 72 hours (the recommended maximum delay per U.S. FDA prescribing information [6]). Since the minimum treatment delay was not reported, we arbitrarily assumed a left-truncation point of 2 hours as the minimum time it would take to obtain LNG-EC after sex. The truncated Weibull distribution provided a substantially better fit to the data based on Bayesian Information Criteria and was chosen to model the distribution of treatment delays in all other example studies.

**3.2 Maximum Effectiveness of LNG-EC in 1-hour Treatment Delays**

Because the discrete nature of the Wilcox pregnancy probabilities, the solution to Eq. (A.1) is in 24-hour units. We used linear interpolation within each 24-hour period to obtain $E_{max,J}$ in 1-hour intervals when computing the weighted-averages of maximum effectiveness values.

**4.0 Data Sources**

Only studies where sufficient detail was provided to estimate the distribution of treatment delays through 72 hours post-sex were considered. Some additional details of the studies are provided below.

1. Raymond *et al.* [7]: this study reported cumulative 12-month pregnancy rates for subjects randomized to either advanced provision of EC or standard (post-coital) access. For the standard access group, we assumed that treatment could not have been received within 2 hours of sex when estimating the (truncated) Weibull distribution of treatment delay, whereas we assumed no left-truncation in the advanced provision group.
2. Noé *et al.* [3] allowed subjects to be treated with LNG-EC up to 120 hours after sex, but fewer than 2% were treated more than 72 hours after sex, so the study was included in our analyses.
3. 1998 WHO study [21]: this study randomized people to either 2 doses of 0.75 mg LNG at 12-hour intervals or the Yuzpe EC regimen. The effectiveness of EC when taken within 72 hours of sex (85% and 57% in the LNG-EC and Yuzpe groups, respectively) was estimated by the study authors assuming all subjects would have ovulated 14 days prior to their next menses. However, a secondary analysis of the Yuzpe data determined that the expected number of pregnancies in the absence of EC use was 29.5% lower when using a more robust cycle-day method to compute counterfactual risks [14]. Applying this same scaling factor to the LNG-EC group leads to an estimated effectiveness of 60%, as noted in Table 1.
4. Ho and Kwan [20]: counterfactual risks of pregnancy tabulated in this manuscript were based on an earlier set of pregnancy probabilities that differ from Wilcox *et al*.
5. Leung et al. [27]: the maximum treatment delay was not reported for this retrospective cohort study. Based on the mean (standard deviation) of 26 hours (19.3), however, most all people ingested EC within 72 hours, and the study was included in our analysis.
